# Urinary mitochondrial DNA level as a biomarker of tissue injury in non-diabetic chronic kidney diseases

**DOI:** 10.1186/s12882-018-1178-9

**Published:** 2018-12-19

**Authors:** Zhongping WEI, Bonnie Ching-Ha KWAN, Kai Ming CHOW, Phyllis Mei-Shan CHENG, Cathy Choi-Wan LUK, Ka-Bik LAI, Philip Kam-Tao LI, Cheuk Chun SZETO

**Affiliations:** Carol & Richard Yu Peritoneal Dialysis Research Centre, Departmentof Medicine & Therapeutics, The Chinese University of Hong Kong, Shatin, Hong Kong China

**Keywords:** Renal failure, Kidney disease, Survival

## Abstract

**Background:**

Urinary mitochondrial DNA (mtDNA) fragment level has been proposed as a biomarker of chronic kidney disease (CKD). In this study, we determine the relation between urinary mtDNA level and rate of renal function deterioration in non-diabetic CKD.

**Methods:**

We recruited 102 non-diabetic CKD patients (43 with kidney biopsy that showed non-specific nephrosclerosis). Urinary mtDNA level was measured and compared to baseline clinical and pathological parameters. The patients were followed 48.3 ± 31.8 months for renal events (need of dialysis or over 30% reduction in estimated glomerular filtration rate [eGFR]).

**Results:**

The median urinary mtDNA level was 1519.42 (inter-quartile range 511.81–3073.03) million copy/mmol creatinine. There were significant correlations between urinary mtDNA level and baseline eGFR (*r* = 0.429, *p* < 0.001), proteinuria (*r* = 0.368, p < 0.001), severity of glomerulosclerosis (*r* = − 0.537, p < 0.001), and tubulointerstitial fibrosis (*r* = − 0.374, *p* = 0.014). The overall rate of eGFR decline was − 2.18 ± 5.94 ml/min/1.73m^2^ per year. There was no significant correlation between the rate of eGFR decline and urinary mtDNA level. By univariate analysis, urinary mtDNA level predicts dialysis-free survival, but the result became insignificant after adjusting for clinical and histological confounding factors.

**Conclusion:**

Urinary mtDNA levels have no significant association with the rate of renal function decline in non-diabetic CKD, although the levels correlate with baseline renal function, proteinuria, and the severity of histological damage. Urinary mtDNA level may be a surrogate marker of permanent renal damage in non-diabetic CKD.

## Background

Chronic kidney disease (CKD) is a global public health issue [1]. The prevalence of CKD is estimated to be 8 to 16% worldwide [2]. A considerable proportion of CKD patients eventually progresses to dialysis-dependent end stage renal disease (ESRD), which is an important economic burden to the health care system [1]. Given the increasing prevalence of CKD, the associated burden of CKD is heavy and ever-rising worldwide [3, 4].

CKD is characterized by progressive renal function loss irrespective to the primary cause of kidney damage [5]. However, the underlying mechanism of progressive renal function loss in CKD, it still remains incompletely understood. Emerging evidence shows that mitochondrial dysfunction plays an important role in this regard, especially in the setting of diabetic nephropathy [6–8]. Glomerular injury or intra-glomerular hypertension results in proteinuria, which is taken up by renal tubular cells, resulting in the generation of reactive oxygen species and cellular oxidative stress [9, 10]. The end result is mitochondrial damage, which contributes to the tubular cell damage as well as epithelial-mesenchymal transition (EMT) [11, 12]. In experimental models of CKD, aborting mitochondrial dysfunction prevents renal tubular cell EMT [9] and renal fibrosis [13].

It has long been known that extracellular cell-free mitochondrial DNA (mtDNA) could easily be detected in various body fluids. Previous studies indicated that mtDNA is released into the systemic circulation after mitochondria is damaging [14, 15]. In addition to blood, dysfunctioned mitochondria in renal tubular cells are also found to release mtDNA into urine, and extracellular mtDNA in urine has recently been explored as the biomarker of various kidney diseases [16–21]. Our previous study showed that urinary mtDNA levels are associated with the severity of renal impairment and histological scarring in diabetic kidney disease [22]. In the present study, we determine the relation between urinary mtDNA level and kidney damage in non-diabetic CKD.

## Methods

### Patient selection

This is an observational study approved by the Clinical Research Ethics Committee of the Chinese University of Hong Kong. All study protocols and procedures were in compliance with the Declaration of Helsinki. We recruited patients with non-diabetic CKD. In short, CKD was defined as estimated glomerular filtration rate (eGFR) ≤60 ml/min/1.73m^2^ or proteinuria ≥1 g/day that persisted for over 3 months. Diabetes was excluded by two fasting plasma glucose tests. A whole-stream early morning urine specimen was collected after written informed consent. Other clinical, biochemical, and histological information were collected by chart review. The eGFR was calculated by the Modification of Diet in Renal Disease (MDRD) formula [23] and the stage of CKD was defined according to the Kidney Disease Improving Global Outcomes (KDIGO) criteria [24].

### Urine mtDNA preparation

Urine specimens were were processed immediately after collection according to methods described previously [25]. Briefly, protease inhibitors were added, and the samples were centrifuged at 1000-g. The supernatant was then stored at − 80 °C until mtDNA extraction and purification by the QIAamp Blood DNA Midi Kit (Qiagen, Valencia, CA).

Urinary mtDNA level was then measured by digital polymerase chain reaction (PCR) by the chip-based dPCR platform (QuantStudio 3D digital PCR System, Life Technologies, Carlsbad, CA). The method of quantification of urinary mtDNA has been described previously [26]. Briefly, extracted DNA was mixed with digital PCR master mix, assay primers and probe. This PCR mix was then loaded on the digital PCR 20 K chip using the QuantStudio 3D digital PCR Chip Loader. PCR reaction was performed using flat-block ProFlex PCR system according to manufacturer instructions. Each PCR run was performed with a blank control by adding water in place of template. After the thermal cycling, the chips were imaged using the QuantStudio 3D digital PCR Chip Reader and analyzed by the QuantStudio 3D Analysis Suite software according the manufacturers’ instruction.

### Morphometric study of kidney biopsy

The degree of renal scarring was assessed by morphometric study on frozen renal biopsy specimen with the Jones’ silver staining. The method of morphometric study has been described previously [27, 28]. In essence, it was a semi-quantitative method using computerized image analysis. The Leica Twin Pro image analysis system (Leica Microsystems, Wetzlar, Germany) was used. Ten glomeruli and 10 randomly selected tubulointerstitial areas were assessed for each patient, and the average percentage of scarred areas were computed.

### Outcome assessment

Recruited patients were followed for at least 24 months. During the follow-up, clinical management of the patients was decided by individual nephrologist and not affected by the study. Serum creatinine, eGFR, and spot urine protein-to-creatinine ratio, were assessed at least every 4 months. The rate of GFR decline was calculated by the least squares regression method. The primary end point was the slope of GFR decline. Secondary outcomes was dialysis-free survival and renal event-free survival. Renal event was defined as over 30% reduction in eGFR as compared to baseline or the need of dialysis.

### Statistical analysis

The software package of SPSS for Windows version 18.0 (SPSS Inc., Chicago, IL) was use to perform the statistical analysis. Continuous data were expressed as mean ± SD or median (inter-quartile range [IQR]) as appropriate. Groups were compared by Chi-square test, independent Student’s t test, Kruskal-Wallis test, or one-way analysis of variance (ANOVA) as appropriate. Spearman’s rank correlation coefficient was use to explore the relation between urinary mtDNA level and clinical or pathological parameters. Urinary mtDNA levels were further sub-grouped to tertiles for further analysis. For renal survival, Kaplan-Meier plot and Cox regression analysis was performed. In addition to urinary mtDNA levels, we used baseline clinical (including eGFR and proteinuria) and histological data (including percentage of glomerulosclerosis and tubulointerstitial fibrosis) as confounding parameters for model construction. These criteria were chosen as covariate because they are well established prognostic markers of CKD. A *P* value below 0.05 was considered statistically significant. All statistical tests report two-tailed probabilities.

## Results

We recruited 102 patients with non-diabetic CKD; 43 had kidney biopsy which showed non-specific nephrosclerosis. The other 59 had CKD of unknown etiology. Table 1 summarizes their demographic, baseline clinical, and biochemical information. All patients received maximal tolerated dose of angiotensin-converting enzyme inhibitor or angiotensin receptor blocker. For the patients with kidney biopsy performed, the median percentage of degree of glomerulosclerosis was 9% (IQR 0 to 55%), and median tubulointerstitial fibrosis 10% (IQR 0 to 30%). There is no statistical significant difference in baseline demographic or clinical data between patients with and without kidney biopsy, except that patients with renal biopsy had a lower serum creatinine and higher urinary mtDNA level than those without biopsy (see Table 1). Mitochondrial DNA was detectable in all urine supernatants, and the median level was 1519.42 million copy per mmol creatinine [IQR 511.81 to 3073.03]. Patients with kidney biopsy had higher urinary mtDNA and lower serum creatinine levels as compared to patients without kidney biopsy (see Table 1).

**Table 1:** Baseline Demographic and Clinical Data

	all cases	with renal biopsy	without renal biopsy	*P* value
no. of case	102	43	59	
sex (M:F)	46: 56	17: 26	29: 30	*p* = 0.4^b^
age (years)	55.07 ± 14.99	55.43 ± 14.87	54.81 ± 15.19	*p* = 0.8^a^
blood pressure (mmHg)				
systolic	151.3 ± 47.0	144.0 ± 19.2	156.7 ± 59.2	*p* = 0.2^a^
diastolic	78.5 ± 13.4	77.7 ± 12.7	79.2 ± 13.9	*p* = 0.6^a^
serum creatinine (μmol/l)	203.50 (98.25–387.75)	127.00 (69.50–283.00)	242.00 (116.00–473.50)	*p* = 0.011^c^
estimated GFR (ml/min/1.73m^2^)	44.27 ± 42.63	53.49 ± 39.90	37.55 ± 43.62	*p* = 0.06^a^
proteinuria (g/day)	1.11 (0.33–2.44)	1.21 (0.61–2.32)	1.03 (0.28–2.45)	*p* = 0.6^c^
urinary mtDNA	1519.42 (511.81–3073.03)	1825.33 (753.63–6749.94)	1207.37 (499.45–2319.48)	*p* = 0.047^c^

*GFR* glomerular filtration rate, *mtDNA* mitochondrial DNA (expressed as million copy per mmol creatinine in urine)

Data are expressed as mean ± standard deviation or median (inter-quartile range [IQR]), and compared by ^a^Student’s t test, ^b^Chi-square test, or ^c^Mann-Whitney U test

### Relation with baseline parameters

**Fig. 1:**
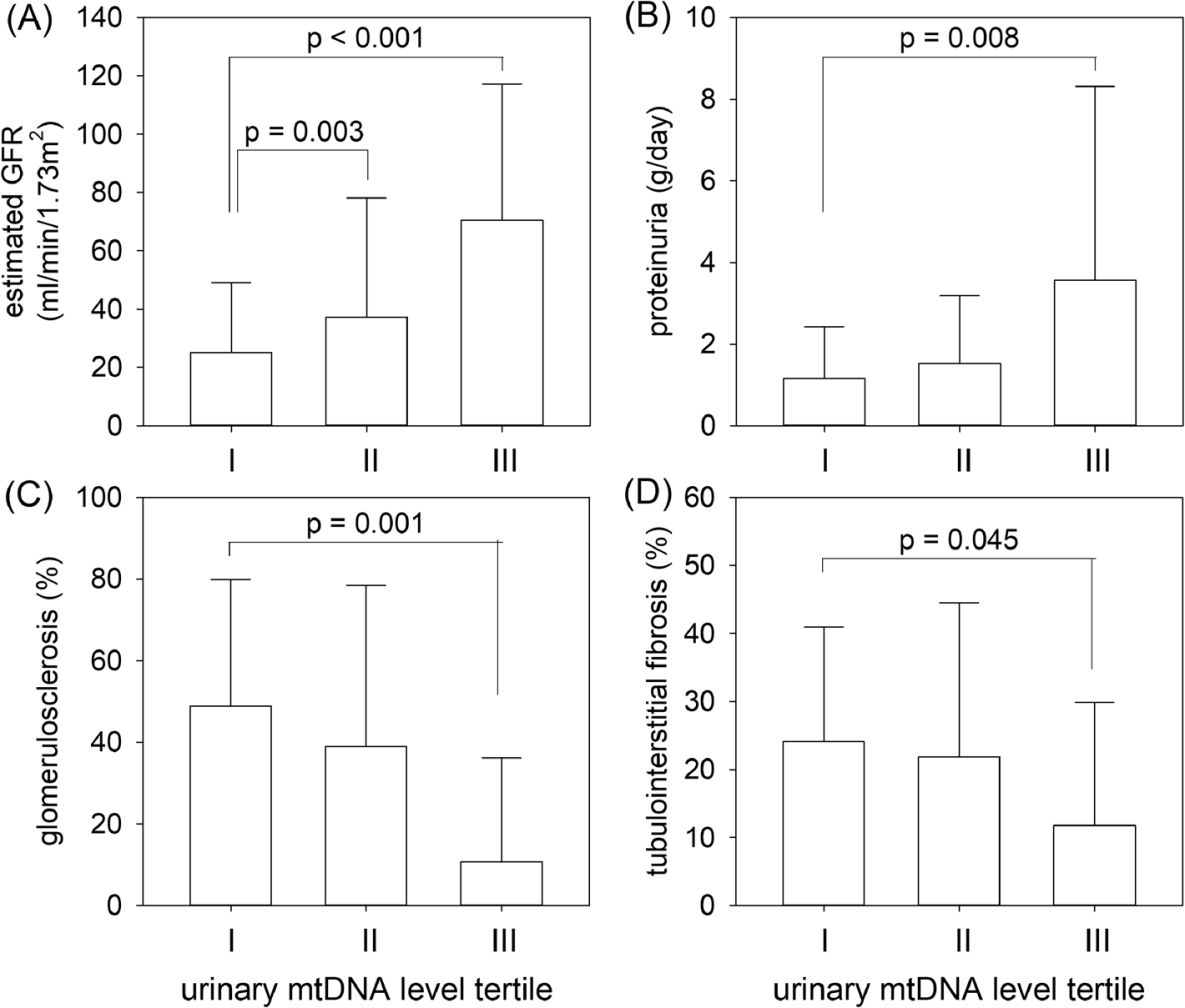
The relation between urinary mitochondrial DNA (mtDNA) level tertiles and (**a**) estimated glomerular filtration rate (GFR); **b** proteinuria; **c** percentage of glomerulosclerosis; and (**d**) tubulointerstitial fibrosis. Tertile I had the lowest while tertile III the highest urinary mtDNA level. Error bars denote standard deviations. Overall comparison by one way analysis of variance (ANOVA). *P* value in figures denote post hoc comparisons by unpaired Student’s t test with Bonferroni’s adjustment for multiple comparison

Urinary mtDNA level had a modest but statistically significant correlation with baseline serum creatinine (*r* = − 0.435, *p* < 0.001), proteinuria (*r* = 0.368, *p* < 0.001), and eGFR (*r* = 0.429, *p* < 0.001) (Fig. 1). The correlation was similar between patients with and without kidney biopsy (details not shown). There were also significant correlations between urinary mtDNA level and percentage of glomerulosclerosis (*r* = − 0.537, *p* < 0.001) and tubulointerstitial fibrosis (*r* = − 0.374, *p* = 0.014).

### Relation with renal function decline

**Fig. 2:**
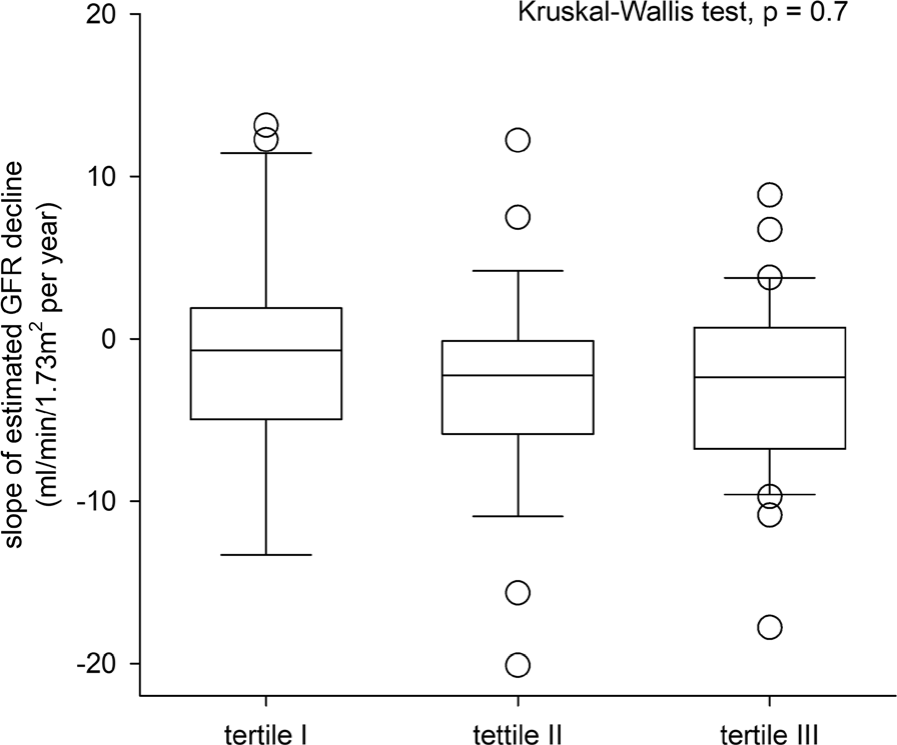
The relation between urinary mitochondrial DNA (mtDNA) level tertiles and the rate of estimated glomerular filtration rate (GFR) decline. Tertile I had the lowest while tertile III the highest urinary mtDNA level. Whisker-box plot, with boxes indicate median, 25th and 75th percentiles, whiskers indicate 5th and 95th percentiles. Data are compared by Kruskal Wallis test

The patients were followed for 48.3 ± 31.8 months. The overall rate of eGFR decline was − 2.18 ± 5.94 ml/min/1.73m^2^ per year. The rate of eGFR decline was similar between patients with and without kidney biopsy (− 1.66 ± 5.01 vs − 2.59 ± 6.61 ml/min/1.73 m2 per year, *p* = 0.5). There was no significant correlation between urinary mtDNA level and the overall rate of eGFR decline (*r* = 0.166, *p* = 0.12). There was no significant difference in the rate of eGFR decline between urinary mtDNA tertiles (Kruskal-Wallis test, *p* = 0.7) (Fig. 2).

**Fig. 3:**
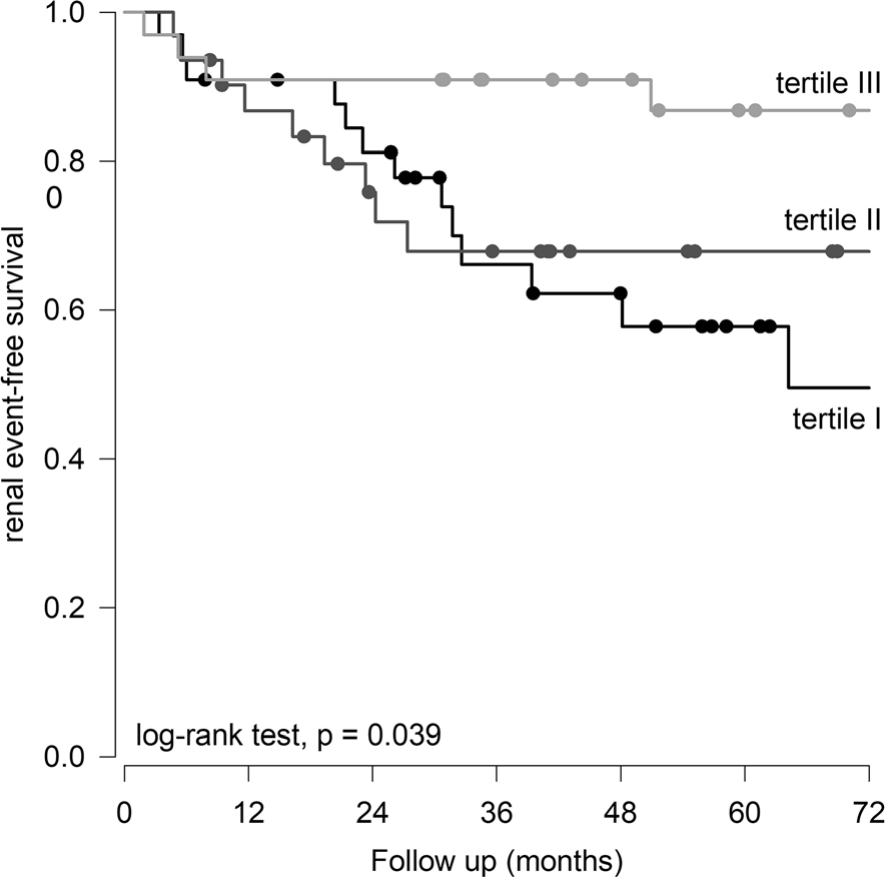
Kaplan-Meier plot of event-free survival. Patients were divided to tertiles of urinary mitochondrial DNA (mtDNA) level. Tertile I had the lowest while tertile III the highest urinary mtDNA level. Data are compared by log rank test

During the study period, 31 patients progress to dialysis dependent renal failure, and another 30 patients had eGFR declined for over 30% from the baseline level. The Kaplan-Meier plot of renal survival is shown in Fig. 3. The dialysis-free survival of urinary mtDNA tertile groups were 56.1, 61.9 and 84.2% at 60 months, respectively (log rank test, *p* = 0.039). Univariate Cox analysis showed that urinary mtDNA level was a predictor of dialysis-free survival (unadjusted hazard ratio [HR] for each log increase in urinary mtDNA, 0.410, 95%CI 0.233–0.721, *p* = 0.002). However, after adjusting for clinical and histological confounding factors with multivariate Cox regression analysis, urinary mtDNA level was not an independent predictor of dialysis-free survival. In this model, only baseline eGFR was an independent predictor of dialysis-free survival (adjusted HR 0.881, 95%CI 0.811–0.958, *p* = 0.003), while urinary mtDNA level had no noticeable effect (adjusted HR 0.804, 95%CI 0.154–4.188, *p* = 0.8). The result remained similar when renal event-free survival was used for analysis (details not shown).

## Discussion

In this study, we show that mtDNA is easy to detect in the urinary supernatant of patients with non-diabetic CKD. Urinary mtDNA level correlates with the degree of renal dysfunction and histological damage. Although urinary mtDNA levels are associated with renal outcome by univariate analysis, the result was not statistically significant after adjusting for clinical and pathological confounders. The role of urinary mtDNA level as a biomarker of patients with non-diabetic CKD may be limited.

Intrarenal oxidative stress plays a critical role in the initiation and progression of kidney disease [29]. The re-absorbing process in renal tubule is highly energy consuming, so that intra-renal hypoxia is an early manifestation of kidney injury [30]. The end result is mitochondrial dysfunction and damage, which release mtDNA into the extracellular space and systemic circulation. In this regard, Chien et al. [31] previously showed that diabetic patients had significantly less cellular mtDNA content, and diabetic atherogenesis is associated with decreased amounts of mitochondrial DNA. Mishra et al. [32] further showed a strong relation between peripheral blood mtDNA damage and diabetic retinopathy, and circulating mtDNA level is a noninvasive biomarker of diabetic retinopathy.

In recent years, urinary cell-free mtDNA level has been explored as a biomarker of kidney diseases. Several studies in the setting of acute kidney injury (AKI) showed that urinary mtDNA level is increased in mice [26] and human [33] after renal ischemic injury, and the level of urinary cell-free mtDNA correlates with the duration of ischemia. Published data on urinary cell-free mtDNA level in the setting of CKD are few. Our previous study showed that reduction of intra-renal mtDNA level in diabetic nephropathy correlates with the degree of renal damage [22], suggesting that intra-renal mitochondrial dysfunction is an important feature of diabetic nephropathy. In another study on biopsy-proved hypertensive nephrosclerosis and IgA nephropathy, we found that urinary mtDNA level correlates with the rate of renal function decline and predicts the risk of doubling of serum creatinine or need of dialysis [34]. In the present study, we extend our observation to non-diabetic kidney diseases with no specific pathological diagnosis.

Our present study showed a significant correlation between urinary mtDNA level and serum creatinine as well as proteinuria. On the first glance, the result may seem counter-intuitive because higher eGFR is expected to be associated with lower proteinuria. However, a lower eGFR probably indicates more permanent renal parenchymal damage and therefore a lower capability to leak mtDNA into the urine. Among the patients with renal biopsy, we observed significant inverse correlations between urinary mtDNA level and the severity of pathological scarring, both in terms of glomerulosclerosis and tubulointerstitial fibrosis. The result is expected because, with increasing fibrosis and declining number of renal parenchymal cells, it is logical to predict that the amount of mtDNA that could be leaked into the urine would reduce. In other words, urinary mtDNA level could be considered as a surrogate marker of the degree of permanent renal parenchymal damage or tissue fibrosis, which also explains its relation with renal survival. Since histological assessment of renal fibrosis is both invasive and subjected to sampling error, our result suggests that urinary mtDNA level may serve as a valuable biomarker of renal damage.

The result of this study is somewhat different from our previous ones [22, 34]. In our previous study on diabetic nephropathy [22], urinary mtDNA levels inversely correlate with eGFR and positively with tubulointerstitial fibrosis. In another study, urinary mtDNA levels positively correlate with proteinuria and inversely with decline of eGFR as well as renal survival in patients with hypertensive nephrosclerosis and IgA nephropathy [34]. The pattern of correlation of our present study is different from the previous one on diabetic nephropathy [22]. Although the underlying reason is not clear, it is possible that high blood glucose causes metabolic stress and directly triggers the damage to tubular epithelial cells, resulting in the excretion of mitochondrial fragments to the extracellular space and urine. Multiple studies showed that hyperglycemia affects renal tubular cells by several mechanisms: overproduction of reactive oxygen species (ROS), activation of apoptotic pathway, and initiation of autophagy [35–39]. Mitochondria, the powerhouse of all cells, are the center of these events. Urinary mtDNA level is conceivably a surrogate marker of mitochondrial repair and regeneration.

There are other limitations of our study. First, the proportion of patients with biopsy is small, limiting the statistical power of multivariate analysis. There may also be unrecognized selection bias for patients to undergo kidney biopsy. Further studies with a larger sample size from an unselected cohort would be necessary to validate our result. Furthermore, the variability of urine mtDNA level is substantial, which may limit the clinical application of urinary mtDNA for risk stratification of CKD.

In conclusion, our study shows that mtDNA is readily detectable in the urinary supernatant of non-diabetic CKD patients. Urinary mtDNA levels have no significant association with the rate of renal function decline in non-diabetic CKD, although the levels correlate with baseline renal function, proteinuria, and the severity of histological damage. Urinary mtDNA level may be a surrogate marker of permanent renal damage in non-diabetic CKD.

## Conclusions

In this study, we find that urinary mtDNA levels have no significant association with the rate of renal function decline in non-diabetic CKD, although the levels correlate with baseline renal function, proteinuria, and the severity of histological damage. Urinary mtDNA level may be a surrogate marker of permanent renal damage in non-diabetic CKD.
